# Electronic Noise Measurement of a Magnetoresistive Sensor: A Comparative Study

**DOI:** 10.3390/s25196182

**Published:** 2025-10-06

**Authors:** Cristina Davidaș, Elena Mirela Ștețco, Liviu Marin Viman, Mihai Sebastian Gabor, Ovidiu Aurel Pop, Traian Petrișor

**Affiliations:** 1 Applied Electronics Department, Technical University of Cluj-Napoca, 400114 Cluj-Napoca, Romania; cristina.davidas@ael.utcluj.ro (C.D.); elena.stetco@ael.utcluj.ro (E.M.Ș.); liviu.viman@ael.utcluj.ro (L.M.V.); 2 Centre for Superconductivity, Spintronics and Surface Science, Technical University of Cluj-Napoca, 400114 Cluj-Napoca, Romania; mihai.gabor@phys.utcluj.ro; 3 European University of Technology, European Union; 4 Physics and Chemistry Department, Technical University of Cluj-Napoca, 400114 Cluj-Napoca, Romania; 5 EUt+ Institute of Nanomaterials & Nanotechnologies—EUTINN, European University of Technology, European Union

**Keywords:** giant magnetoresistance, low-frequency noise, low-noise amplifiers

## Abstract

The intrinsic noise of giant magnetoresistive (GMR) sensors is large at low frequencies, and their resolution is inevitably significantly limited. Investigation of GMR noise requires the use of measurement systems that have lower noise than the sample. In this context, the main purpose of this study is to evaluate the effectiveness of two electronic noise measurement configurations of a single GMR sensing element. The first method connects the sample in a voltage divider configuration and the second method connects in a Wheatstone bridge configuration. Three amplification set-ups were investigated: a low-noise amplifier, an ultra-low-noise amplifier and an instrumentation amplifier. Using cross-correlation, the noise of the measurement system introduced by the amplifiers was reduced. Noise spectra were recorded at room temperature in the frequency range of 0.5 Hz to 10 kHz, under different sample bias voltages. The measurements were performed in zero applied magnetic field and in a field corresponding to the maximum sensitivity of the sensor. From the noise spectra, the detectivity of the sensor was determined to be in the 200–300 nT/√Hz range. Good agreement was observed between the results obtained using all three set-ups, suggesting the effectiveness of the noise measurement systems applied to the magnetoresistive sensor.

## 1. Introduction

The intrinsic noise of a sensor is one of the main obstacles in low-field applications, limiting the signal-to-noise ratio (SNR) and ultimately the measurement sensitivity [1]. Giant magnetoresistive (GMR) and tunnel magnetoresistive (TMR) structures exhibit larger low-frequency 1*/f* noise compared to anisotropic magnetoresistive (AMR) sensors [2,3]. This limits the minimum detectable field, i.e., the detectivity of the sensors. Noise level thus becomes a key aspect in choosing a sensor for a specific application [4]. Accordingly, the experimental measurement of electronic noise, especially the 1*/f* component, is an essential tool for sensor performance optimization.

Therefore, dedicated measurement systems characterized by the low level of the background noise are necessary to accurately estimate the noise spectrum. A solid body of work exists on the noise evaluation of magnetoresistive sensors using various noise measurement techniques. However, to date, there is a lack of comprehensive reports on the design and fabrication of amplification systems and the measurement configuration used to characterize magnetoresistive sensors. The main purpose of this paper is an in-depth analysis and comparison of two noise evaluation methods that are typically used in the literature: the voltage divider and Wheatstone bridge configurations. Through a tutorial-like approach, we describe in detail the implementation of both configurations from design and fabrication to their validation by measuring the noise spectrum of an in-house fabricated GMR sensor. The background noise performance of the presented systems is comparable to some of the best reports in the literature [5,6,7].

The first method involves biasing the sample in a voltage divider configuration [5,6,7,8,9,10] and amplifying its noise signal using a low-noise amplifier. Two amplification set-ups were implemented based on the design reported in [11]: a low-noise amplifier (LNA) and an ultra-low-noise amplifier (ULNA). The main difference between the two designs is their respective background noise levels. The equivalent input noise voltage of ULNA is lower than that of LNA. In the second method, the GMR sample is connected in a Wheatstone bridge configuration [12,13,14,15,16,17,18,19,20,21,22,23,24,25,26]. The GMR sensor was connected in a quarter bridge configuration in which the sensor represents one arm of the bridge. This differs from the half bridge and full bridge configuration [22], where two and four sensors are employed. The advantage of such a configuration is that only one sensing element is needed for performing the noise measurements. The upper arms consisted of ballast resistors with much larger resistance values than those of the sample resistance. Unlike the voltage divider configuration, as will be shown in Section 3.2.1, connecting the sample to a Wheatstone bridge allows the measurement of only the 1*/f* noise component. Cross-correlation was used in all instances to reduce measurement system noise. Amplifier design and component selection were carefully considered to obtain the optimum noise performance of the measurement set-ups. We show that the investigated noise measurement methods can be effectively used for the accurate evaluation of the intrinsic noise generated by the GMR sensor. The remainder of this paper is structured as follows: Section 2 presents the details of the experimental implementation of our study, Section 3 is dedicated to an extensive analysis of the noise generated by each part of the measurement configuration, Section 4 presents the measurement results for the set-up amplification and background noise, respectively, the sensor transport properties and sensor noise, and Section 5 is devoted to the conclusions.

## 2. Materials and Methods

**Figure 1:**
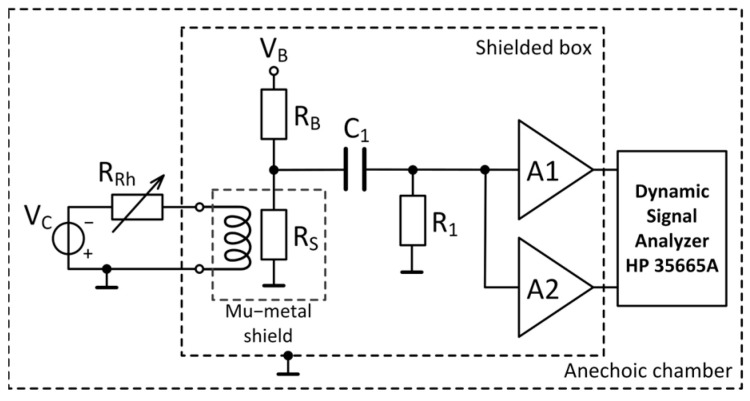
Noise measurement set-up for the voltage divider configuration.

**Figure 2:**
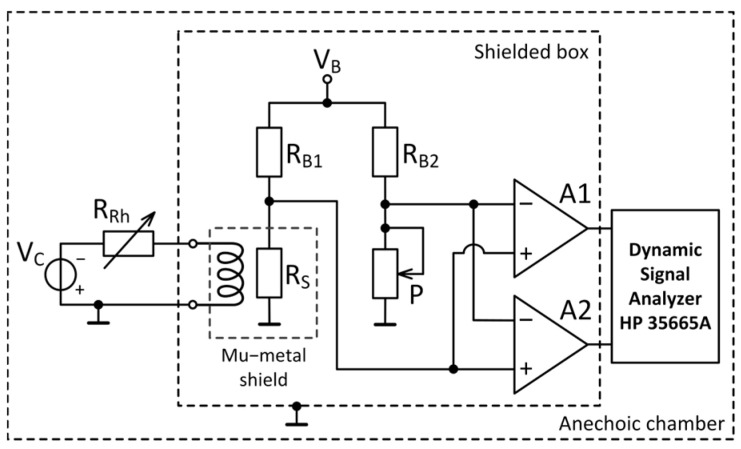
Noise measurement set-up for the Wheatstone bridge configuration.

A two-channel cross-correlation method is implemented to measure the noise of a giant magnetoresistive sensor. The cross-correlation procedure results in the suppression of the noise sources generated by two identical amplifiers (A1 and A2 in Figure 1 and Figure 2) which produce uncorrelated effects at the outputs of the two channels. Due to the reduction in the background noise contribution of the amplifiers, the noise spectrum density of the GMR sensor may be accurately measured. The sample had a multilayer spin valve structure with the following layer sequence: Si//Ta(2.3 nm)/NiFe(2.0 nm)/CoFe(1.0 nm)/Cu(2.0 nm)/CoFe(2.0 nm)/IrMn(8.0 nm)/Ta(2.3 nm) [27]. The magnetoresistive element was patterned in a meander shape with two measurement contacts. Two sensor-biasing configurations were investigated for noise measurements at room temperature: a voltage divider configuration (Figure 1) and a Wheatstone bridge configuration (Figure 2). The cross-spectrum of the sample was measured using an HP 35665A spectrum analyzer in the frequency range of 0.5 Hz to 10 kHz, applying a Hanning time window. 

**Figure 3:**
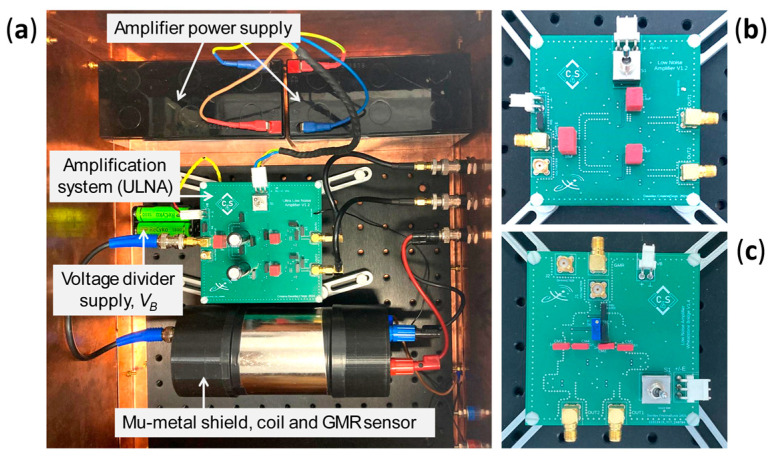
(**a**) Experimental set-up of the noise measurement system in the voltage divider configuration using the ULNA, (**b**) LNA and (**c**) Wheatstone bridge boards.

The sensor voltage and amplifier bias were supplied by batteries to minimize noise. The GMR sensor and coil for generating the magnetic field were enclosed in a double-walled mu-metal cylinder to increase the immunity to external electromagnetic fields. The coil is supplied by a 12 V rechargeable battery (*V_c_*) and the potentiometer (*R_Rh_*) determines the current through it. The measurement system was placed in a copper box, and measurements of the GMR sensor noise were performed in an anechoic chamber to suppress the pickup of environmental noise, Figure 3.

In addition, special attention has been dedicated to enhancing the immunity of circuits boards to external interference signals. This is usually performed to ensure that the circuits function reliably, even in noisy environments, where electromagnetic interference (EMI) or other external signals can disrupt their operation. In this context, the following techniques were used:

- *Grounding.* The PCB stackup was structured into four layers. The inner layers were dedicated to the power and ground planes. The ground plane reduces electromagnetic interference pickups. To minimize radiated emission [28], the top and bottom layers were filled with a solid ground plane around the signals. A continuous ground plane across the PCB reduces the loop area for signals, and correctly routing the ground with minimal resistance and inductance provides a path for EMI dissipation.
- *Shielding.* The sample was connected to the inputs of the two identical and symmetrical stages of the voltage amplifier. To limit the perturbation of useful signals due to the emission of interfering signals, a shielding (guarding) technique was implemented [29]. The purpose of guarding is to ensure that sensitive signals, such as analog signals, are less susceptible to interference, thereby ensuring a more reliable and accurate performance of the PCB. The procedure is realized with stitching vias (0.3 mm drill) connected to the ground plane and placed on both sides of the susceptible traces on a 1.27 mm grid. The entire surface of the PCB was shielded by grounded vias (2.54 mm grid) that acted as a Faraday cage enclosure.
- *Layout Optimization.* The passive components were placed very close to the ICs, which minimized parasitic capacitance. The inputs and feedback loops were routed as short as possible with rounded corners to avoid reflection [30]. The voltage amplifiers have a high common-mode rejection ratio (CMRR > 100 dB) to ensure the amplification of the noise signal of the sample and the attenuation of the unwanted common-mode signals. The output voltage of the instrumentation amplifiers was measured with respect to the potential of the reference pin, which was connected to the ground plane through a via placed close to it.
- *Decoupling.* To provide a stable supply voltage and prevent spikes on the power supply line of the ICs, 22 μF tantalum capacitors with a low ESR value (100 mΩ) were used. The decoupling capacitors were placed near the power supply pins of the operational amplifiers and routed using a short trace. The amplifiers operate with a 24 V supply voltage and a typical 4 mA quiescent current. Under these conditions, the junction temperature reaches 30 °C. To improve the thermal management, the junction temperature was reduced to the ambient temperature by soldering the exposed pad of the ADA4625 (Analog Devices) operational amplifier to the ground plane. Thermal vias placed on the thermal pad were used to decrease the chip junction temperature and ensure good thermal dissipation.

The PCB layer stack-up of all three amplification systems is given in the Supplementary Materials.

Specific component selection for the amplification systems was performed keeping in mind the following input specifications: a bandwidth covering the low-frequency part of the spectrum, <1 Hz–100 kHz, an amplification to allow for a reliable determination of the noise level, 60 dB, and a background noise spectral density that is lower than the sample thermal noise, <6 nV/√Hz, to allow for the accurate measurement of the sample noise.

## 3. Noise Analysis of the Measurement Configurations

In the following paragraphs, we present a detailed analysis of the noise generated by each component of the measurement systems. All noise models described below have noise sources marked as voltage and current fluctuations (*v_n_*, *i_n_*). Noise is expressed and calculated as voltage spectral density (VSD), *e_n_,* or current spectral density, *i_n_*.

### 3.1. Voltage Divider Configuration

#### 3.1.1. Voltage Divider

**Figure 4:**
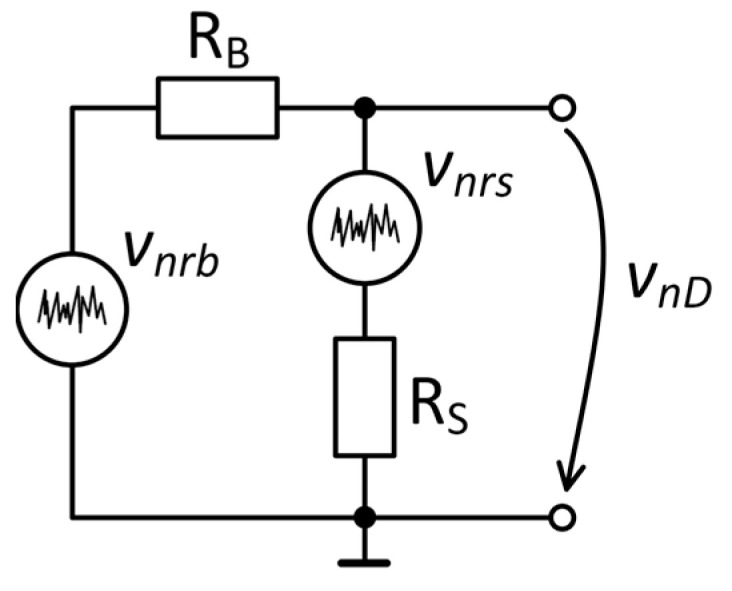
Noise model of the voltage divider.

The noise of the GMR sensor, *R_S_*, was measured by applying a DC voltage across a resistor *R_B_* connected in series with the sample, as shown in Figure 1. Applying the superposition theorem for the noise model presented in Figure 4 and assuming all noise sources to be uncorrelated, the noise across the device under test is as follows [31]:

(1) $$e_{nD}=\sqrt{{\left(\frac{R_{S}}{R_{B}+R_{S}}\right)}^{2}{e_{nrb}}^{2}+{\left(\frac{R_{B}}{R_{B}+R_{S}}\right)}^{2}{e_{nrs}}^{2},}$$

where *e_nrb_* is the VSD of the series resistance and *e_nrs_* is the VSD of the sample. For large values of series resistance (*R_B_* ≫ *R_S_*), the VSD *e_nD_* coincides with the noise of the sample. A value of 100 kΩ was selected for *R_B_* to obtain an equivalent resistance that is equal to the resistance of the GMR sensor used in our study, 2.26 kΩ in zero magnetic field. A low 1*/f* noise metal foil resistor was chosen such that it did not influence the noise evaluation of the sensor. Its expected very low noise level translates into a noise index of less than −30 dB [32,33,34,35].

#### 3.1.2. *R*_1_*C*_1_ Filter

**Figure 5:**
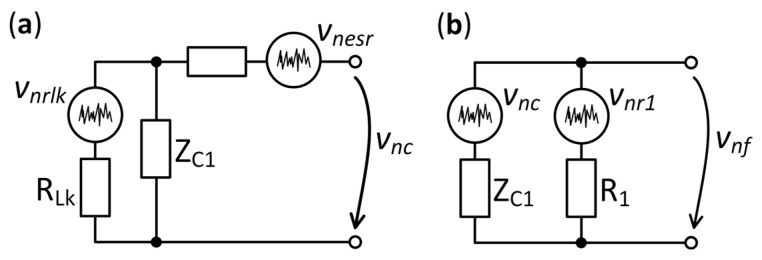
Noise model of (**a**) capacitor *C*_1_, and (**b**) the *R*_1_*C*_1_ filter.

The DC voltage at the terminals of the sample was rejected using an *R*_1_*C*_1_ AC coupling filter, as shown in Figure 1. The noise of the capacitor is given by its leakage resistance *R_Lk_* and equivalent series resistance *R_ESR_*. The noise model of the capacitor is shown in Figure 5a and includes noise sources that correspond to parasitic elements. The VSD of the output fluctuation *v_nc_* is given by the following equation:

(2) $$e_{nc}=\sqrt{{e_{nesr}}^{2}+\frac{{e_{nrlk}}^{2}}{1+{\left(\frac{f}{f_{0}}\right)}^{2}}},$$

where *e_nesr_* is the thermal VSD of the *R_ESR_*, *e_nrlk_* is the thermal VSD of the *R_Lk_* and *f*_0_ is the cutoff frequency of the *R_Lk_C*_1_ filter:

(3) $$f_{0}=\frac{1}{2\pi R_{Lk}C_{1}}.$$

**Figure 6:**
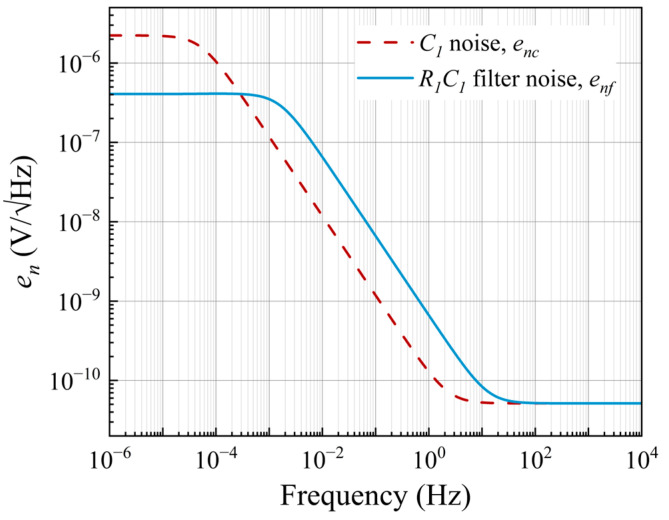
Voltage spectral density of the capacitor *C*_1_, and the AC coupling filter *R*_1_*C*_1_.

In this study, a polyester capacitor with a capacitance of 10 μF was chosen. Its leakage resistance has a high value of *R_Lk_* = 300 MΩ, while the equivalent series resistance is *R_ESR_* = 160 mΩ. The dotted line in Figure 6 shows the response curve of the filter with a cutoff frequency *f*_0_ = 53 μHz. Above *f*_0_, the contribution of *e_nrlk_* is significantly attenuated by the low capacitive reactance. At high frequencies, the noise density corresponds to the thermal noise of *R_ESR_* (51.5 pV/√Hz at *f* > 1 kHz). This value represents the noise level of the filter stopband, and consequently, the lower limit of the background noise of the measurement system.

The *R*_1_*C*_1_ AC coupling filter has the noise model shown in Figure 5b. Resistor *R*_1_ generates thermal noise with a VSD, *e_nr_*_1_. The VSD of the filter *e_nf_* is as follows:

(4) $$e_{nf}=\sqrt{\frac{{\left(\frac{f}{f_{c}}\right)}^{2}}{1+{\left(\frac{f}{f_{c}}\right)}^{2}}{e_{nc}}^{2}+\frac{{e_{nr1}}^{2}}{1+{\left(\frac{f}{f_{c}}\right)}^{2}}},$$

where *f_c_* is the cutoff frequency of the *R*_1_*C*_1_ filter:

(5) $$f_{c}=\frac{1}{2\pi R_{1}C_{1}}.$$

The calculated *e_nf_* voltage noise characteristic for a value of *R*_1_ = 10 MΩ is shown in Figure 6. The influence of the value of *R*_1_ on *e_nf_* was presented in [31], without considering *R_ESR_* (see text below). Below *f_c_*, the thermal noise of *R*_1_ is dominant. In the high-frequency domain, the thermal noise voltage *e_nr_*_1_ is shunted by the capacitor *C*_1_. Thus, the lowest noise level for all the curves is given by the thermal noise of *R_ESR_*. A value of 10 MΩ was chosen for *R*_1_ because of its negligible noise contribution in the measurement frequency range, 1.3 nV/√Hz at 0.5 Hz.

#### 3.1.3. Low-Noise Amplifier

**Figure 7:**
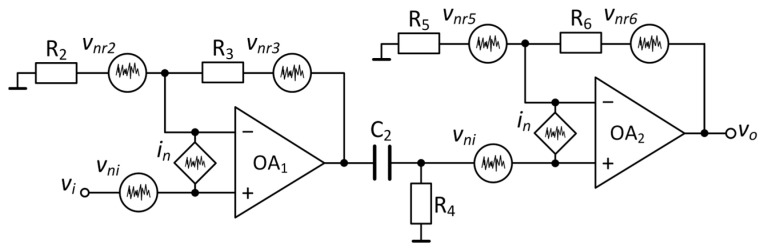
Noise model of a single channel of the low-noise amplifier.

The noise signal generated by the sample is amplified using a low-noise amplifier based on the ADA4625 op-amps [11], as shown in Figure 7. The op-amps have an input voltage noise, *e_ni_*, of 3.3 nV/√Hz, and an input current noise, *i_n_*, of 4.5 fA/√Hz at 1 kHz. Every channel uses a non-inverting amplifier with a gain of 40.09 dB, followed by a second amplifier with a 20.83 dB amplification. The output offset voltage of the first amplifier is eliminated by the *R*_4_*C*_2_ filter (*R*_4_ = 2.2 MΩ, *C*_2_ = 6.8 μF) with a cutoff frequency of 10.6 mHz [31]. The electric circuit diagram of the low-noise amplifier is given in the Supplementary Materials. To minimize the thermal noise of the network resistors, the circuit was designed according to the following conditions: (a) the value of the resistances must be in the range of tens of ohms to hundreds of ohms; (b) *R*_2_ ≪ *R*_3_, *R*_5_ ≪ *R*_6_, so the parallel resistances *R_p_*_1_ ≈ *R*_2_ and *R_p_*_2_ ≈ *R*_5_, where *R_p_*_1_ is the parallel combination of *R*_2_ and *R*_3_, and *R_p_*_2_ is the parallel combination of *R*_5_ and *R*_6_, respectively. In this way, the thermal noise of parallel resistances may be ignored, as it is smaller than the intrinsic voltage noise of the operational amplifier (√(4*k_B_TR_p_*_1_) ≪ *e_ni_* and √(4*k_B_TR_p_*_2_) ≪ *e_ni_*).

Another aspect is related to the current noise of the operational amplifiers, *i_n_*, that flows through the parallel resistances, establishing a noise VSD that is reflected in the input of the amplification system. The superposition theorem for the noise model is applied. Assuming that all noise sources are uncorrelated, the equivalent input noise of each amplification stage can be expressed as follows:

(6) $$e_{nA1}=\sqrt{{e_{ni}}^{2}+{\left(i_{n}\cdot R_{p1}\right)}^{2}},$$

(7) $$e_{nA2}=\sqrt{{e_{ni}}^{2}+{\left(i_{n}\cdot R_{p2}\right)}^{2}},$$

where *e_nA_*_1_ is the input-referred noise of the first amplification stage and *e_nA_*_2_ is the input-referred noise of the second amplification stage, respectively. The last terms in the above equations have noise levels of 45 fV/√Hz and 41 fV/√Hz, respectively. This is significantly lower than the input voltage noise density of the operational amplifier ADA4625; therefore, the impact of current noise can be ignored.

Finally, the total input-referred noise of the amplification system is calculated as follows [31]:

(8) $$e_{nA}=\sqrt{{e_{ni}}^{2}+{\left(\frac{e_{ni}}{A_{1}}\right)}^{2}},$$

where *A*_1_ is the gain of the first amplification stage.

The noise of the first voltage amplifier is dominant and has a value of 3.3 nV/√Hz, which corresponds to the input voltage noise of the operational amplifier.

#### 3.1.4. Ultra-Low-Noise Amplifier

**Figure 8:**
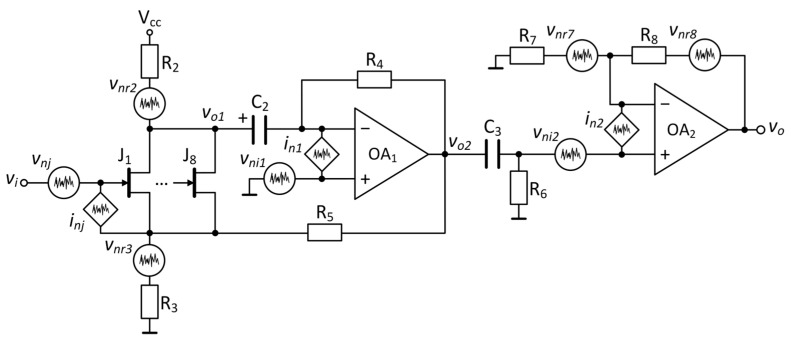
Noise model of a single channel of the ultra-low-noise amplifier.

This configuration is based on a transimpedance amplifier coupled to a common-source JFET amplifier, resulting in a high input impedance hybrid amplifier [11], shown in Figure 8. The input impedance increases due to negative feedback to the source terminal of the common-source JFET configuration. The offset voltage at the output of the hybrid amplifier is eliminated by the second AC coupling filter, *R*_6_*C*_3_ (*R*_6_ = 2.2 MΩ, *C*_3_ = 6.8 μF) with a cutoff frequency of 10.6 mHz. The sample noise is amplified by a second stage, connected in series with the first stage, consisting of a non-inverting amplifier based on a low-noise operational amplifier. The electric circuit diagram of the ultra-low-noise amplifier is given in the Supplementary Materials. In the following paragraphs, we present an analysis of the noise generated at each stage of the amplification system.

The first analysis is of the common-source amplifier based on the JFET 2SK3557 (onsemi, Phoenix, AZ, USA). The circuit provides a high input impedance front-end stage for a voltage amplifier based on a low-noise operational amplifier (OA1). Thermal and shot noise were considered for the analysis. Shot noise is generated by the DC reverse saturation gate current (gate cutoff current), *I_GSS_*. Its noise current spectral density, *i_nj_*, is given by the following equation [36,37,38,39]:

(9) $$i_{nj}=\sqrt{2qI_{GSS}},$$

where *q* is the electron charge (1.6 × 10^−19^ C). Based on the datasheet value, *I_GSS_* = 1 nA, so the shot noise was calculated to have a negligible value of 18 fA/√Hz.

Thermal noise is generated by the Brownian motion of charge carriers in the channel of the device at equilibrium. Its noise VSD, *e_nj_* is given by the following equation [38,40]:

(10) $$e_{nj}=\sqrt{4k_{B}TR_{ch}}=\sqrt{4k_{B}T\left(\frac{2}{3g_{m}}\right)},$$

where *k_B_* is the Boltzmann constant (1.38 × 10^−23^ J/K), *T* is the absolute temperature, *R_ch_* is the resistance of the channel, and *g_m_* is the small-signal transconductance. From Equation (10), it is apparent that a high *g_m_* leads to a decrease in the thermal noise of the JFET channel. *g_m_* has the largest value when the device operates with a drain current, *I_D_*, very close to the value of the drain-to-source saturation current, *I_DSS_*. To further increase the value of *g_m_*, eight identical JFETs were connected in parallel [11]. In agreement with the noise model of a parallel-stage amplifier [39], the equivalent input noise voltage was reduced by a factor of √N, where N is the number of JFETs connected in parallel.

**Figure 9:**
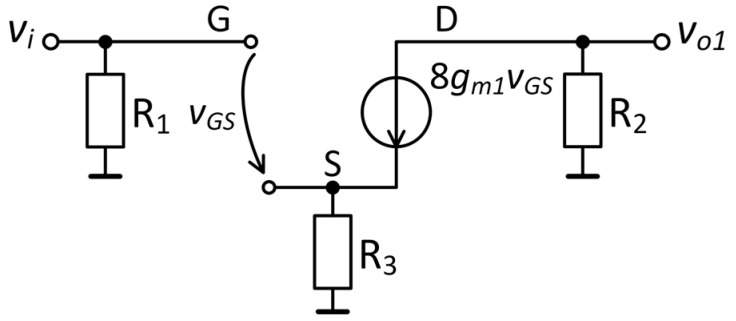
Small-signal model of the common-source amplifier.

The small-signal model of the common-source amplifier is illustrated in Figure 9. The input signal *v_i_* is applied to the gate of the transistor, whereas the output signal *v_o_*_1_ is taken from the drain terminal. The gain of the common-source amplifier, *A*_1_, is expressed as follows:

(11) $$A_{1}=\frac{v_{o1}}{v_{i}}=-\frac{8g_{m1}R_{2}}{1+8g_{m1}R_{3}},$$

where *R*_2_ is the drain resistance, *R*_3_ is the source resistance and *g_m_*_1_ is the small-signal transconductance of only one JFET of the eight transistors connected in parallel. The parameter *g_m_*_1_ has the following form:

(12) $$g_{m1}=\frac{1}{\sqrt{8}}\frac{2\sqrt{I_{D}I_{DSS}}}{\left|V_{th}\right|},$$

where *I_D_* is the total drain current, *I_DSS_* is the drain-to-source saturation current for a configuration with only one JFET device, and *Vth* is the threshold voltage. By calculating the small-signal transconductance *g_m_*_1_ from Equation (12), a value of 24 mS was obtained for an *I_D_* of 41.6 mA. Finally, amplification *A*_1_ in the passband is 24.32 dB.

**Figure 10:**
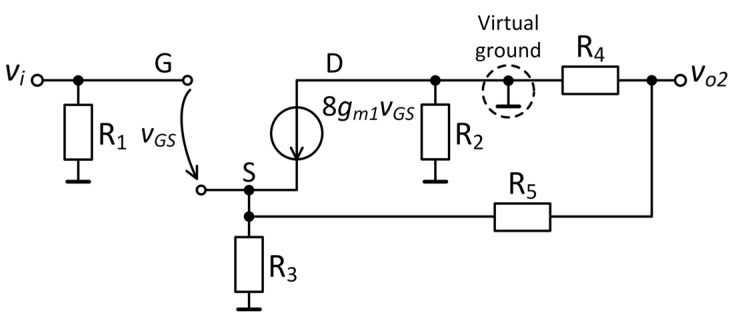
Small-signal model of the hybrid amplifier.

The common-source amplifier was coupled to a transimpedance amplifier using a DC-blocking capacitor *C*_2_. Resistors *R*_5_ and *R*_3_ set the closed-loop gain, reducing the variations in the amplification due to the transconductance of the JFETs [41,42]. The transimpedance amplifier is based on the low-noise operational amplifier LT1028 (Analog Devices, Wilmington, MA, USA), OA1, with an input voltage noise, *e_ni_*_1_, of 0.85 nV/√Hz and an input current noise, *i_n_*_1_, of 1 pA/√Hz at 1 kHz. An analysis of the small-signal model is presented in Figure 10, which is limited to the high-frequency domain for simplicity. Thus, *C*_2_ was replaced by a short-circuit, and the drain resistance *R*_2_ was eliminated because of the virtual ground of the operational amplifier OA1. The gain of the hybrid amplifier, *A*_2_, is as follows:

(13) $$A_{2}=\frac{v_{o2}}{v_{i}}=\left(1+\frac{R_{5}}{R_{3}}\right)\frac{8g_{m1}R_{e}}{1+8g_{m1}R_{e}},$$

where *R*_5_ is the amplification-setting resistor and *R_e_* is the equivalent resistance given by the parallel combination of *R*_3_ and (*R*_4_ + *R*_5_). R_4_ represents the feedback resistor of the transimpedance amplifier. Because *R*_3_ ≪ *R*_4_ and *R*_5_, the value of *R_e_* is approximately equal to the source resistance. The calculated value of amplification *A*_2_ in the passband was 30.5 dB.

The last stage is a simplified non-inverting amplifier using an ADA4625 (OA2) low-noise operational amplifier with a gain of 34.15 dB. To reduce the noise contribution of the network resistors (*R*_7_, *R*_8_), the circuit was designed according to the principles presented in the low-noise amplifier section. Assuming all noise sources to be uncorrelated, the total input referred noise is expressed as follows:

(14) $$e_{nA}=\sqrt{{e_{nA1}}^{2}+{\left(\frac{e_{nA2}}{A_{2}}\right)}^{2}+{\left(\frac{e_{nA3}}{A_{2}}\right)}^{2}},$$

where *e_nA_*_1_ is the noise of the common-source amplifier, *e_nA_*_2_ is the noise of the transimpedance amplifier and *e_nA_*_3_ is the noise of the non-inverting amplifier, respectively.

The expression of the VSD, *e_nA_*_1_, is shown as follows:

(15) $$e_{nA1}=\sqrt{{e_{nj}}^{2}+{\left(\frac{e_{nr2}}{A_{1}}\right)}^{2}+{e_{nre}}^{2}},$$

where *e_nr_*_2_ is the thermal noise of the drain resistance *R*_2_ and *e_nre_* is the thermal noise of the equivalent resistance *R_e_*. Because of the large amplification *A*_1_, the noise generated by the drain resistance *R*_2_ has a small contribution. The first term represents the JFET noise.

The second main noise source from the measurement system corresponds to the transimpedance amplifier, which can be calculated as follows:

(16) $$e_{nA2}=\sqrt{{e_{ni1}}^{2}+{\left(i_{n1}\cdot R_{4}\right)}^{2}}.$$

The VSD of the non-inverting amplifier is as follows:

(17) $$e_{nA3}=\sqrt{{e_{ni2}}^{2}+(i_{n2}\cdot {R_{p})}^{2}},$$

where *R_p_* is the parallel combination of *R*_7_ and *R*_8_. From the above expressions, the noise of the ultra-low-noise amplifier was calculated to be *e_nA_* = 0.5 nV/√Hz in the white, high-frequency (*f* > 100 Hz) domain. If we refer all noise sources to the input of the amplification system, we conclude that the common-source amplifier has the highest contribution to the total noise, with a value of 0.49 nV/√Hz. The noise of the transimpedance amplifier is insignificant, 78.8 pV/√Hz, and the noise of the last voltage amplifier is 99.3 pV/√Hz.

### 3.2. Wheatstone Bridge Configuration

#### 3.2.1. Wheatstone Bridge

**Figure 11:**
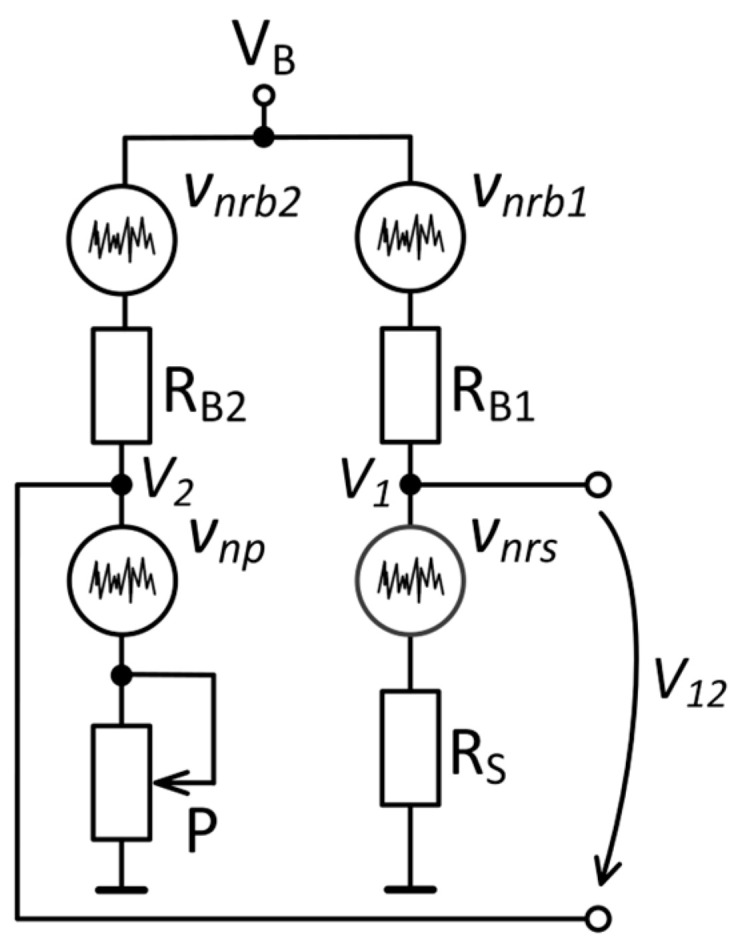
Noise model of the Wheatstone bridge.

The case in which the sample is connected in a Wheatstone bridge configuration is illustrated in Figure 11. An excitation voltage *V_B_* is applied across both arms of the bridge. The upper arms represent the ballast resistors, *R_B_*_1_ and *R_B_*_2_, while the sample is represented by *R_S_*. The bridge output voltage is measured differentially and balanced using a variable resistor *P* (*V*_12_ ≈ 0 V), so only the noise component is measured. The electric circuit diagram of the Wheatstone bridge and corresponding amplification circuit is given in the Supplementary Materials.

The output noise voltage of the Wheatstone bridge, *e_n_*_12_, is the noise contribution generated by both arms:

(18) $$e_{n12}=\sqrt{{e_{n1}}^{2}+{e_{n2}}^{2}},$$

where *e_n_*_1_ and *e_n_*_2_ are the voltage spectral densities of the first and second arms, respectively. The upper arms of the bridge consist of metal foil resistors with *R_B_*_1_ ≫ *R_S_* and *R_B_*_2_ ≫ *P*. A wire-wound variable resistor for *P* was chosen because of its low 1*/f* noise [32,34]. The noise spectrum of the potentiometer was measured in a voltage divider configuration using the ULNA. The potentiometer resistance value was set at 2.26 kΩ, to match the GMR sensor zero-field resistance, while the bias voltage was 719 mV. The voltage noise density at 1 Hz was 6 nV/√Hz, which is close to the corresponding thermal noise, 6.1 nV/√Hz, demonstrating that the potentiometer 1*/f* noise is indeed negligible.

The noise at the terminals of the potentiometer and sample are given by the following equations:

(19) $${e_{n1}}^{2}={\left(\frac{R_{S}}{R_{B1}+R_{S}}\right)}^{2}{e_{nrb1}}^{2}+{\left(\frac{R_{B1}}{R_{B1}+R_{S}}\right)}^{2}{e_{nrs}}^{2},$$

(20) $${e_{n2}}^{2}={\left(\frac{wP}{R_{B2}+wP}\right)}^{2}{e_{nrb2}}^{2}+{\left(\frac{R_{B2}}{R_{B2}+wP}\right)}^{2}{e_{np}}^{2},$$

where *e_nrb_*_1_ is the voltage spectral density of the series resistance *R_B_*_1_, *e_nrb_*_2_ is the voltage spectral density of the series resistance *R_B_*_2_, *e_nrs_* is the voltage spectral density of the sample, *e_np_* is the voltage spectral density of the potentiometer and *w* is a constant in the [0, 1] range.

To characterize the sample noise, *e_nrs_*, it is necessary to perform two separate measurements: one with *V_B_* = 0 V and the other with *V_B_* ≠ 0 V. In the first case, the thermal component of the bridge noise, *e_n_*_12_*^′^*, is measured, whereas in the second case, both 1*/f* and thermal components, *e_n_*_12_^″^, are evaluated. If the flicker noise of the potentiometer is negligible, subtracting the results of the two measurements (*V_B_* = 0 V and *V_B_* ≠ 0 V) results in the elimination of the thermal noise component. Hence, the 1*/f* component of the sample noise, *e_nrs,_*_1*/f*_, is given by the following equation [43]:

(21) $$e_{nrs,\;1/f}=\sqrt{{\left({e_{n12}}^{\prime \prime }\right)}^{2}-{\left({e_{n12}}^{\prime }\right)}^{2}\;}.$$

This is the main difference between the two measurement configurations. The voltage divider measures both the flicker noise and thermal noise of the sensor, whereas the Wheatstone bridge allows the analysis of only the flicker noise.

#### 3.2.2. Instrumentation Amplifier

**Figure 12:**
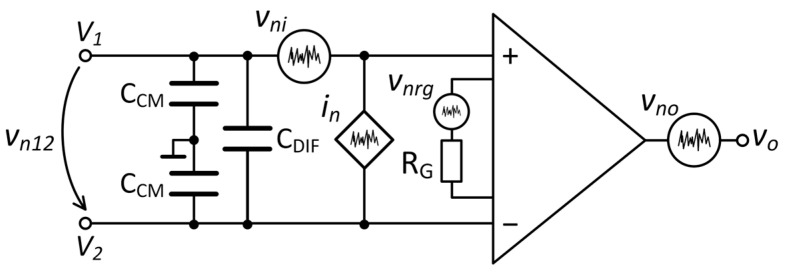
Noise model of the single amplification channel of the instrumentation amplifier.

The Wheatstone bridge was coupled to an LT1167 (Analog Devices) low-noise instrumentation amplifier (INA). The INA is modeled as a noiseless operational amplifier with two equivalent noise sources at the input: a voltage source with spectral density *e_ni_* (7.5 nV/√Hz at 1 kHz) and a noise current source *i_n_* (56 fA/√Hz at 1 kHz), and an equivalent noise source at the output with a spectral density, *e_no_*, of 67 nV/√Hz at 1 kHz (Figure 12). The system amplification is 60 dB, determined by the external gain resistor, *R_G_*, which generates thermal noise, *e_nrg_*.

The total amplifier voltage noise density, *e_nA_*, is given by the following equation:

(22) $$e_{nA}=\sqrt{{e_{ni}}^{2}+{\left(\frac{e_{no}}{A_{v}}\right)}^{2}+{e_{nrg}}^{2}},$$

where *A_v_* is the amplifier voltage gain of the instrumentation amplifier. The noise contribution of the voltage amplifier was calculated to be 7.5 nV/√Hz at 1 kHz.

The total input voltage noise density of the INA, including the current noise source, is expressed as follows:

(23) $$e_{n,RTI}=\sqrt{{e_{nA}}^{2}+{e_{nrs}}^{2}{+\left(i_{n}\cdot R_{S}\right)}^{2}}.$$

**Figure 13:**
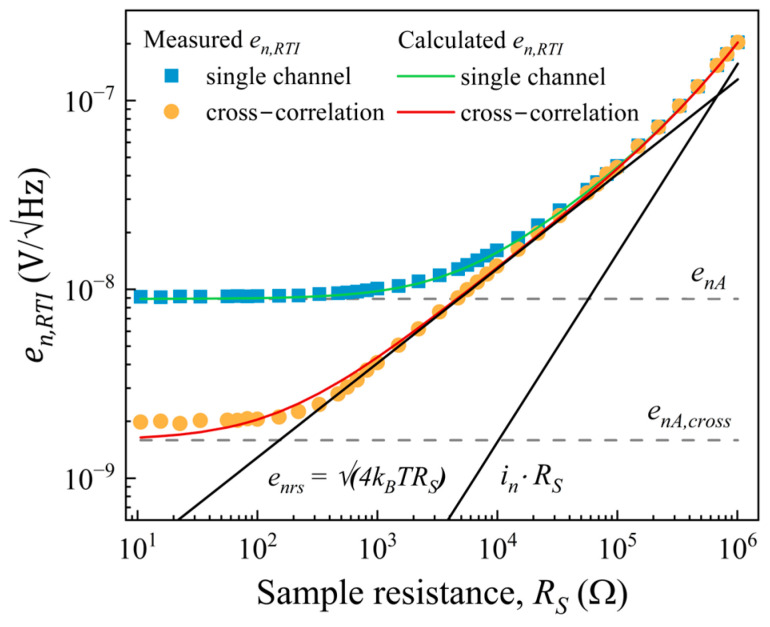
Noise density of the instrumentation amplifier as a function of the sample resistance in single-channel and cross-correlation configurations.

The noise performance of the instrumentation amplifier LT1167 was measured and evaluated using both one amplification channel and cross-correlation. The white background noise of the amplification system has a mean value of 8.9 nV/√Hz that is reduced to 1.59 nV/√Hz by cross-correlation. To verify that the LT1167 amplifier is adapted to the resistance of our GMR device, we measured the total noise voltage density across a wide range of resistances from 10 Ω to 1 MΩ. Plots of the mean voltage noise density as a function of the sample resistance are shown in Figure 13. The theoretical value of the sample thermal noise density, √(4*k_B_TR_S_*), is included in the graph. At low resistance values, *R_S_* < 40 Ω, in the case of cross-correlation, and *R_S_* < 20 kΩ for one amplification channel, *e_n,RTI_* is limited by the input voltage noise of the amplifier, *e_nA_*. As *R_S_* increases, the total noise coincides with the thermal noise of the sample for a wide range of resistances, particularly in the case of cross-correlation measurements. Above 100 kΩ, the current noise, *i_n_*・*R_S_*, becomes the dominant contributor to the total noise, because the voltage noise generated by its flow through the sample is larger than its thermal noise, *i_n_*·*R_S_* > √(4*k_B_TR_S_*). The mean value of the current noise was measured to be *i_n_* = 155 fA/√Hz.

For the GMR sample resistance of 2.26 kΩ, it may be seen that cross-correlation is suitable for measuring its electronic noise. Its thermal noise is well above the amplifier noise level, *e_nA,cross_*, and its resistance value does not introduce significant voltage noise from *i_n_*. In addition, the sample noise voltage was evaluated in the bandpass region of the filter formed by the sample resistance and parasitic capacitance. The sample *R_S_* and the common mode capacitance, *C_CM_*, form a low-pass filter with a cutoff frequency of 44 MHz (*C_CM_* = 1.6 pF) without any impact in the measurement frequency range, 0.5 Hz–10 kHz.

## 4. Results and Discussion

### 4.1. Measurement System Performance

**Figure 14:**
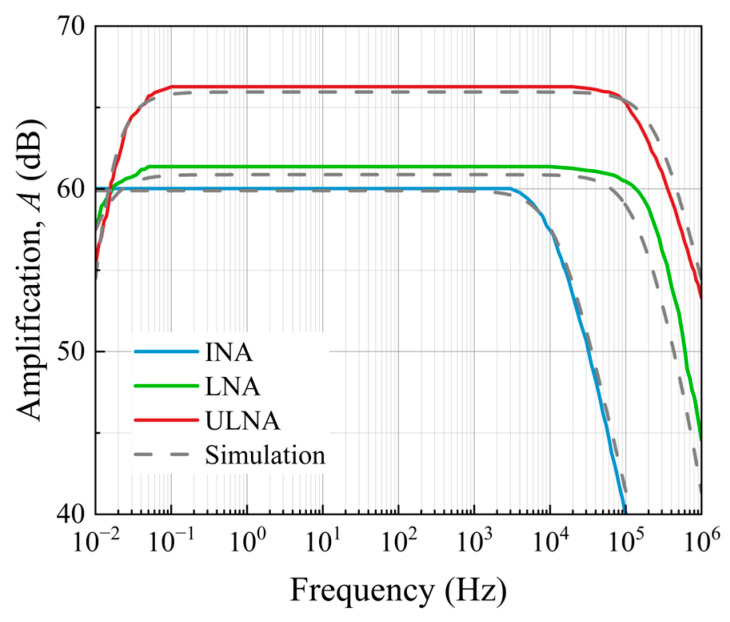
Measured and simulated amplification of the three different systems (INA, LNA [31], ULNA), and their corresponding bandwidths.

Calibration of the amplification for the configurations was performed by measuring the thermal noise of metal film resistors with electrical resistances varying from 68 Ω to 10 kΩ at room temperature in the 1–10 kHz frequency range. The amplification was calculated by dividing the mean of the output noise by the calculated Johnson noise of the resistors. The results are presented in Table 1. The measured frequency responses of the amplifiers are shown in Figure 14. The results are in good agreement with the simulated and calibrated data (Table 1).

**Figure 15:**
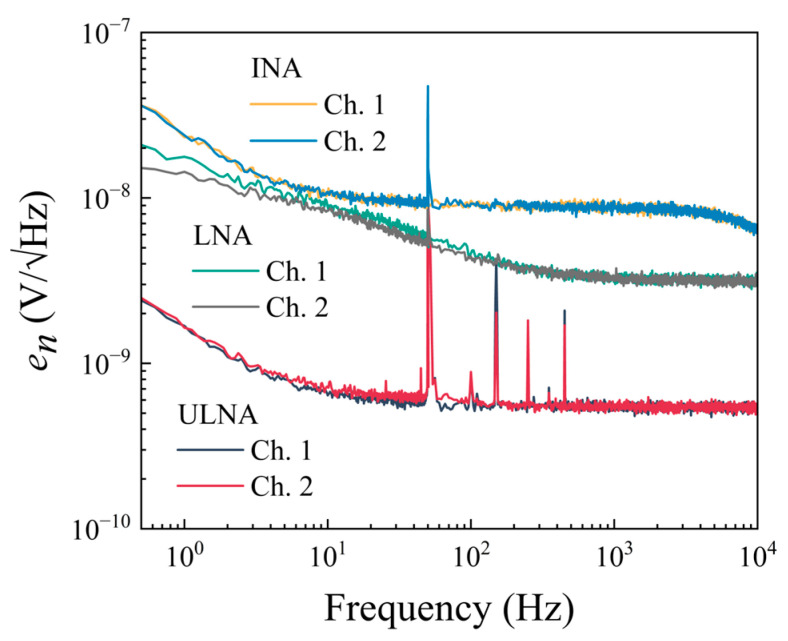
Experimental noise spectra for each channel of the measurement systems.

**Figure 16:**
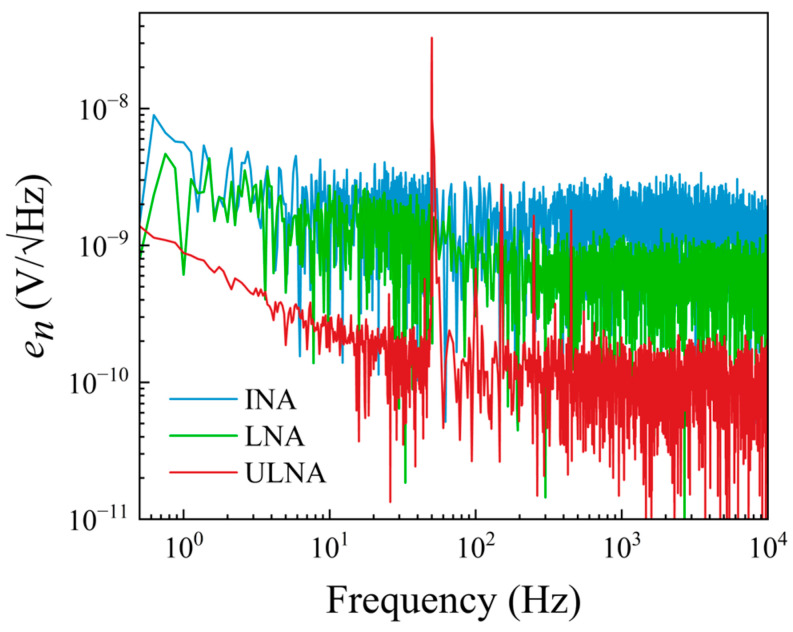
Cross-correlation spectra of the three amplification configurations.

Figure 15 shows the measured noise VSD for each channel of the amplifiers, with both inputs shorted to the ground. The sharp peaks in the noise spectra correspond to the power-line frequency (50 Hz) and harmonics (see, for example, [18,44]). The spectra obtained for each channel of the low-noise amplifier are equal to the calculated voltage noise, 3.3 nV/√Hz, for high frequencies, *f* > 1 kHz. It may be seen in Figure 16 that the background noise decreases from 3.2 nV/√Hz to 0.6 nV/√Hz after cross-correlation. In the case of the ultra-low-noise amplifier, the equivalent input voltage noise decreases from 0.54 nV/√Hz to 0.1 nV/√Hz at *f* > 1 kHz after the cross-correlation due to the common-source configuration with eight JFETs placed in parallel. As expected, the measured spectra of each channel for the instrumentation amplifier coincided with the equivalent input voltage noise of the op-amp, 8.9 nV/√Hz at high frequencies, *f* > 1 kHz. After the cross-correlation, the background noise decreases to 1.59 nV/√Hz.

Table 2 shows values of amplifier noise VSD reported in the literature for various, commonly used amplifiers and amplification systems. The presented data were obtained in the context of measuring magnetoresistive sensor noise. One of the commonly used amplifiers is INA103 (Texas Instruments) [14,45], which has very low noise in cross-correlation measurements, 0.41 nV/√Hz, but also when used in a single channel configuration, approximately 1 nV/√Hz. These values compare to our LNA set-up when used for cross-correlation measurements. For single channel measurements, the noise in our case is significantly higher, 3.2 nV/√Hz. The LT1167 instrumentation amplifier was used for measuring the GMR sensor in a quarter bridge configuration. It has considerably larger noise than the INA103, 8.9 nV/√Hz. However, in the case of INA103, due to its higher current noise density, ∼2 pA/√Hz, it cannot be used to measure high sample resistances, >200 Ω [46]. Since, in our case, the GMR sensor has a zero-field resistance of 2.26 kΩ, LT1167 was chosen. As shown in Figure 13, LT1167 can be used to reliably measure the noise of resistances in the kΩ range. The best results were obtained on the ULNA amplification system, 0.54 nV/√Hz, which shows a similar performance to the amplification systems used by Weitensfelder et al. [47], ∼0.6 nV/√Hz at 100 kHz, based on the differential ultra-low-noise amplifier design proposed by Scandurra et al. [48].

### 4.2. Magnetic Field Response of the GMR Sensor

**Figure 17:**
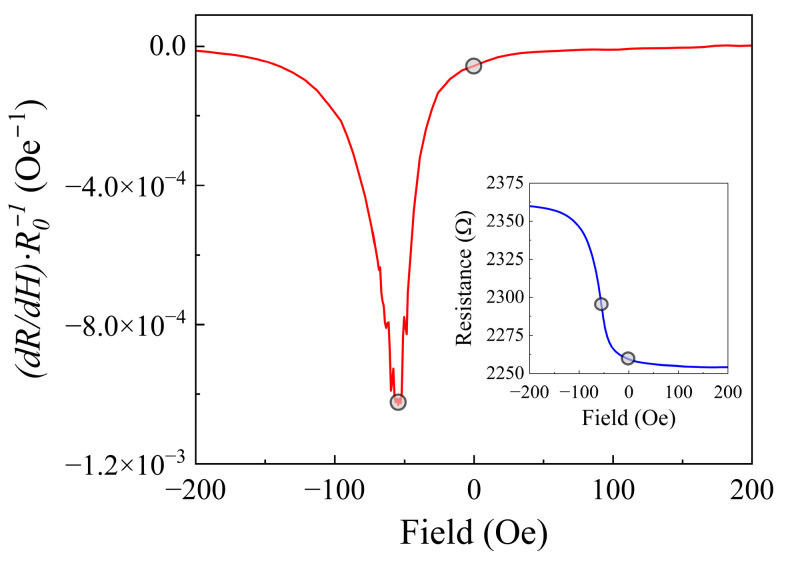
Magnetic field sensitivity of the GMR sensor, and magnetoresistive response to an applied magnetic field (inset).

The three amplification configurations were used to study the electrical noise generated by the GMR spin-valve. The magnetic field response of the sensor is shown in Figure 17 (inset). Its resistance variation corresponds to a magnetoresistive ratio, *MR* of 4.75%, defined as follows:

(24) $$MR\left(\%\right)=\frac{R_{max}-R_{min}}{R_{min}}\times 100\;\left(\%\right),$$

the numerical differentiation of the R(H) curve was used to calculate the sensor sensitivity, *γ_R_*, expressed in Oe^−1^ [47]:

(25) $$\gamma_{R}=\left(\frac{dR}{dH}\right)\cdot \frac{1}{R_{0}},$$

where *R*_0_ is the sensor resistance in a zero applied magnetic field. The results of these calculations are shown in Figure 17. The maximum sensitivity was 1.03 × 10^−3^ Oe^−1^, recorded at a field of −54.6 Oe.

### 4.3. Electronic Noise of the GMR Sensor

**Figure 18:**
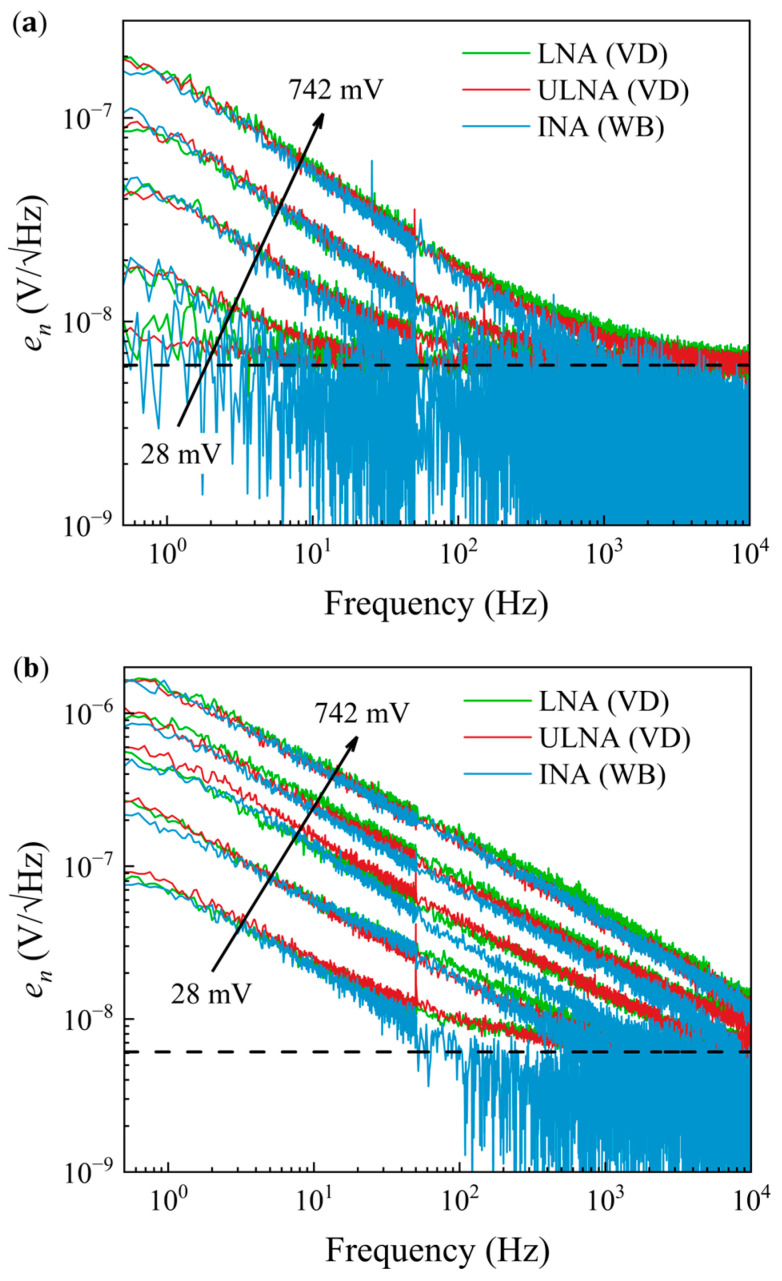
Cross-correlation spectra of the GMR sensor noise in a (**a**) 0 Oe and (**b**) −54.6 Oe applied magnetic field, for different sensor biases. The spectra were recorded using the three amplification systems, LNA, ULNA and INA, in the voltage divider (VD) and Wheatstone bridge (WB) configurations. The dashed line corresponds to the theoretical thermal noise of the sensor.

Voltage noise spectral density measurements (Figure 18) were performed at maximum sensitivity (Figure 18b) and in a zero-field (Figure 18a) for the three amplification set-ups. The GMR sensor was connected in the voltage divider (VD) configuration and its noise was measured using the LNA and ULNAs, and also in a Wheatstone bridge (WB) configuration, in which case, the noise signal was amplified by the INA. Spectra were recorded at different sensor bias voltages in the range of 28–742 mV. No overheating or drift effects were observed during the measurements, as the maximum dissipated power on the sample did not exceed 1 mW [50]. The sensor voltage noise spectral density contains two terms: flicker noise, relevant at low frequencies with a 1*/f* frequency dependence, and thermal, white noise, which is dominant in the higher frequency range, with a constant spectral density. 1*/f* noise is higher in the maximum sensitivity field, as shown in Figure 18b, and increases with the voltage drop across the sensors for both field values. Generally, very good agreement was observed in the 1*/f* part of the spectrum between the measurements performed in both configurations for all amplification schemes. Due to the measurement procedure outlined in Section 3.2, in the case of the Wheatstone bridge, the sample thermal noise is eliminated, leaving only the amplifier noise baseline at higher frequencies. It may be seen in Figure 18 that the high frequency part of the spectrum, with a mean value of 2.8 nV/√Hz above 1 kHz, drops below the theoretical thermal noise of the sensor, 6.1 nV/√Hz, marked by the dashed line, and is closer to the noise floor of the INA, 1.59 nV/√Hz (see Figure 16).

Analytically, *e_n_*^2^, may be written as the sum of 1*/f* and thermal contributions:

(26) $$e_{n}^{2}=\frac{\alpha_{H}}{n\Omega }V^{2}\cdot \frac{1}{f}+4k_{B}TR_{S},$$

where *α_H_* is the Hooge parameter, *n* is the carrier concentration in the sensor, *A* is the sensor area, *V* is the bias voltage of the sensor, and *R_S_* is the resistance of the sensor. The high value of the low-frequency 1*/f* noise component determines the minimum detectable field of the sensor. Thus, the Hooge parameter, *α_H_*, may be regarded as a figure of merit for the sensing performance. It is also used to parameterize 1*/f* noise and has been studied extensively in magnetoresistive sensors, particularly in magnetic tunnel junctions (MTJs) (see, for example, [51]), and in GMR spin valves (see, for example, [52]). A strong correlation exists between *α_H_* and the resistance variation as a function of the applied magnetic field, *dR/dH*. The maximum value of *α_H_* was recorded when *dR/dH* peaked [7]. Thus, from a practical perspective, the minimum detectable field evaluation from electronic noise measurements should be determined in an external field corresponding to the highest sensitivity of the sensor. This was achieved by calculating the sensor field detectivity, expressed in T/√Hz. The detectivity is defined as follows [47]:

(27) $$D=\frac{\sqrt{e_{n}^{2}}}{\gamma_{R}V}.$$

**Figure 19:**
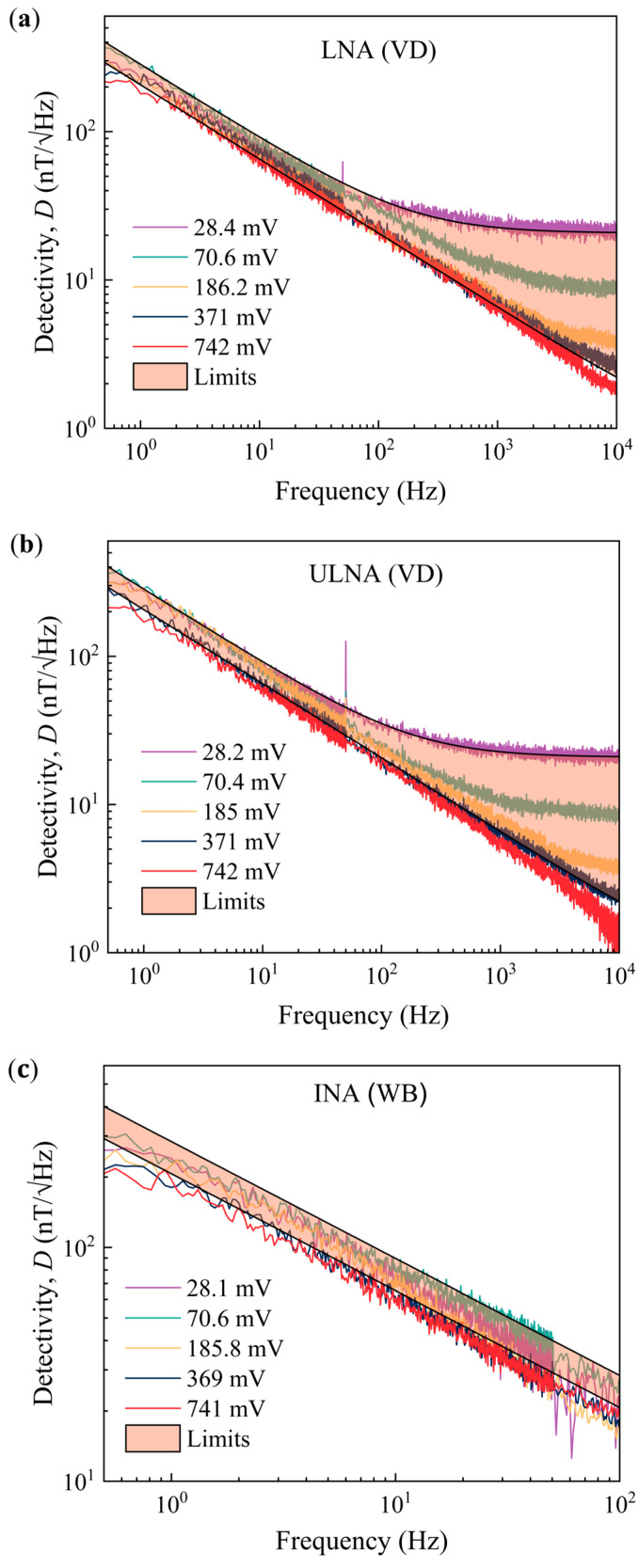
Sensor detectivities, calculated limits and measured data, for the three amplification systems, (**a**) LNA, (**b**) ULNA, and (**c**) INA, and corresponding measurement configurations, the voltage divider (VD) and the Wheatstone bridge (WB).

It was calculated using the *e_n_* data in Figure 18b and sensor field sensitivity, *γ_R_*. The results for each amplification set-up are shown in Figure 19. In the case of the Wheatstone bridge, because the thermal part does not originate from the sensor itself, it has been omitted, and only the 1*/f* part is shown. Scaling of the 1*/f* component in *D* is observed because of the bias voltage dependence of the 1*/f* noise, as shown in Equation (26). The field detectivity of the sensor is situated in the 200–300 nT/√Hz range at 1 Hz. This is a rather high value, single GMR sensors with detectivities of 30 nT/√Hz have been reported [53], that is directly attributed to high 1*/f* noise levels and consequently a high Hooge constant.

The 1*/f* part of the spectrum can be parameterized using the Hooge constant, *α_H_*. The normalized Hooge constant, *α_H_/n*, was calculated by fitting the low-frequency noise spectrum presented in Figure 18 from 1 to 5 Hz with a *C*·*f^−^*^1^ curve. The pre-factor *C* was the only fitting parameter. Knowing the sensor sensitivity, *γ_R_*, and the sample area, *A* = 6.46 × 10^3^ μm^2^, *α_H_/n* was determined to be (4.2 ± 1.3) × 10^−8^ μm^2^. This represents the mean and standard deviation extracted from the values corresponding to all three amplifications and all bias voltages. As expected from the high detectivity value, *α_H_/n* is also higher than other values reported in the literature. Fermon et al. [54] reported a Hooge constant of 7 × 10^−3^ for a GMR spin valve with a structure similar to that presented in our study. This corresponds to an *α_H_/n* value of approximately 1.8 × 10^−11^ μm^2^ (*n* = 4 × 10^8^ μm^−2^, [22]). The Hooge constant may be subjected to large variations due to the magnetic domain structure of the GMR sensor [54]. Our large detectivity values, determined by high 1*/f* noise levels and consequently by a large *α_H_*, may be due to the lack of optimization of the ferromagnetic layers’ anisotropies. The optimization process may include parameters such as, film thickness, annealing temperature and annealing duration. Also, our patterned devices exhibit a degree of lateral roughness. Rough sample edges represent domain wall nucleation sites, which may be the cause of magnetic instability determining higher 1*/f* noise, a large Hooge constant, and the reduced detectivity performance of our sensor. The low-level background noise of our amplifiers ensures that the measurement systems presented are capable of accurately determining the 1*/f* noise of our sample, and are suited for even lower 1*/f* values, such as the ones found in optimized, or commercial sensors.

The normalized Hooge constant, *α_H_/n*, was used to calculate the detectivity of the sensor. The results for all three cases are presented in Figure 19 (shaded area). The spread of the calculated curves was obtained by considering the variation in *α_H_/n* within one standard deviation. Good agreement was found between the calculated and measured detectivities for the three configurations. This confirms that all the employed set-ups are valid candidates for the electronic noise measurement of magnetoresistive sensors.

## 5. Conclusions

In this study, we investigated the use of two measurement configurations for the electronic noise evaluation of a GMR sensor, with three amplification set-ups. A voltage divider configuration with low-noise and ultra-low-noise amplification and a Wheatstone bridge configuration with an instrumentation amplifier were studied. Detailed noise modeling was presented for all components of the set-ups. Cross-correlation measurements effectively eliminated the amplification circuit noise, allowing a sub nV/√Hz baseline noise density in the case of the ultra-low-noise amplifier. Noise level performance measurements indicated that all configurations are adapted for the study of our GMR sensor noise. Fitting the 1*/f* part of the noise spectra allowed the determination of the normalized Hooge constant, *α_H_/n*, which in turn was used to calculate the sensor detectivity, *D*. Detectivity was determined to be in the 200–300 nT/√Hz range. It exhibited the expected voltage scaling behavior in all measured configurations. Good agreement was found between the measured and calculated detectivities. Accordingly, it was demonstrated that all three set-ups were suitable for the noise characterization of the GMR sensor. A distinctive feature of the Wheatstone bridge measurement set-up is that it requires an additional measurement step, that eliminates the thermal noise of the investigated sample, leaving only its 1*/f* component. Our study suggests that both the voltage divider and Wheatstone bridge can be used to evaluate de 1*/f* noise of a single element sensor. However, this is true if the noise of the sensor is higher than that of the amplifiers. For lower noise sensors, the voltage divider configuration with the ultra-low-noise amplifier is the best solution for electronic noise evaluation.

## Figures and Tables

**Table 1:** Amplification of the measurement systems. The amplification values are shown for experimental (Exp.), simulation (Sim.), and calibrated (Cal.) results.

Amplifier	Passband	Amplification (Exp., Sim., Cal.)		
INA	DC—11 kHz	60 dB	59.88 dB	59.9 dB
LNA [31]	12 mHz–220 kHz	61.36 dB	60.87 dB	60.64 dB
ULNA	24 mHz–178 kHz	66.27 dB	65.95 dB	65.88 dB

**Table 2:** Comparison between reported amplifier noise voltage spectral density in the literature and in the present work.

Reference	Sample	Amplifier	VSD (Single Channel)	VSD (Cross-Correlation)
Stutzke et al. [21]	AMR, GMR, TMR	not specified	4.4 nV/√Hz	0.7 nV/√Hz
Jonker et al. [14]	magnetoresistive	INA103	1.3 nV/√Hz	0.41 nV/√Hz
		Brookdeal 5004	0.86 nV/√Hz	
Pannetier-Lecoeur et al. [45]	GMR	INA103	1.2 nV/√Hz	
Allegre et al. [20]	GMR	INA163	1 nV/√Hz	
Liu et al. [49]	TMR	JFET IF3602, LT1028	3.1 nV/√Hz	
Weitensfelder et al. [47]	GMR, TMR	JFET IF3602, INA131	~0.6 nV/√Hz	
This work:				
INA	GMR—bridge	LT1167	8.9 nV/√Hz	1.59 nV/√Hz
LNA	GMR—divider	ADA4625	3.2 nV/√Hz	0.6 nV/√Hz
ULNA	GMR—divider	JFET 2SK3557, LT1028, ADA4625	0.54 nV/√Hz	0.1 nV/√Hz

## Data Availability

The original contributions presented in this study are included in the article. Further inquiries can be directed to the corresponding author(s).

## Supplementary Materials

The following are available online at https://www.mdpi.com/article/10.3390/s25196182/s1: Figure S1: Low-noise amplifier; (**a**) Circuit diagram; (**b**) PCB stack-up. Figure S2: Ultra-low-noise amplifier; (**a**) Circuit diagram; (**b**) PCB stack-up. Figure S3: Wheatstone bridge and amplification circuit; (**a**) Circuit diagram; (**b**) PCB stack-up.

## Abbreviations

The following abbreviations are used in this manuscript:

GMR	Giant magnetoresistive
LNA	Low-noise amplifier
ULNA	Ultra-low-noise amplifier
INA	Instrumentation amplifier
VD	Voltage divider
WB	Wheatstone bridge
VSD	Voltage spectral density
