# Supplementary material for: Electronic Noise Measurement of a Magnetoresistive Sensor: A Comparative Study
Source: Sensors (Basel). 2025 Oct 6;25(19):6182. doi: 10.3390/s25196182 (PMC12527107; doi:10.3390/s25196182)

Supplementary Material

The electric circuit diagrams and the corresponding PCB layer stack-ups are provided for the three amplification systems presented in the article.

Figure S1. Low-noise amplifier

(**a**) Circuit diagram

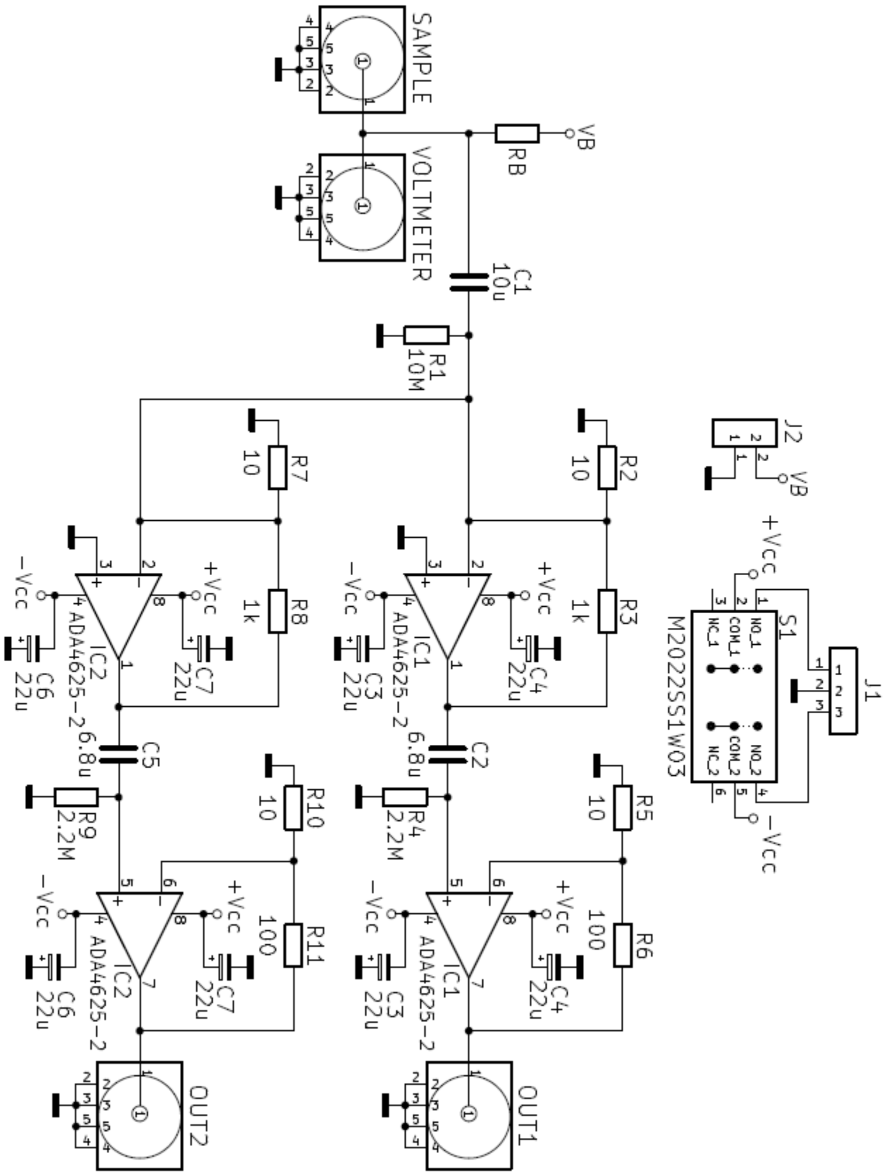

(**b**) PCB stack-up

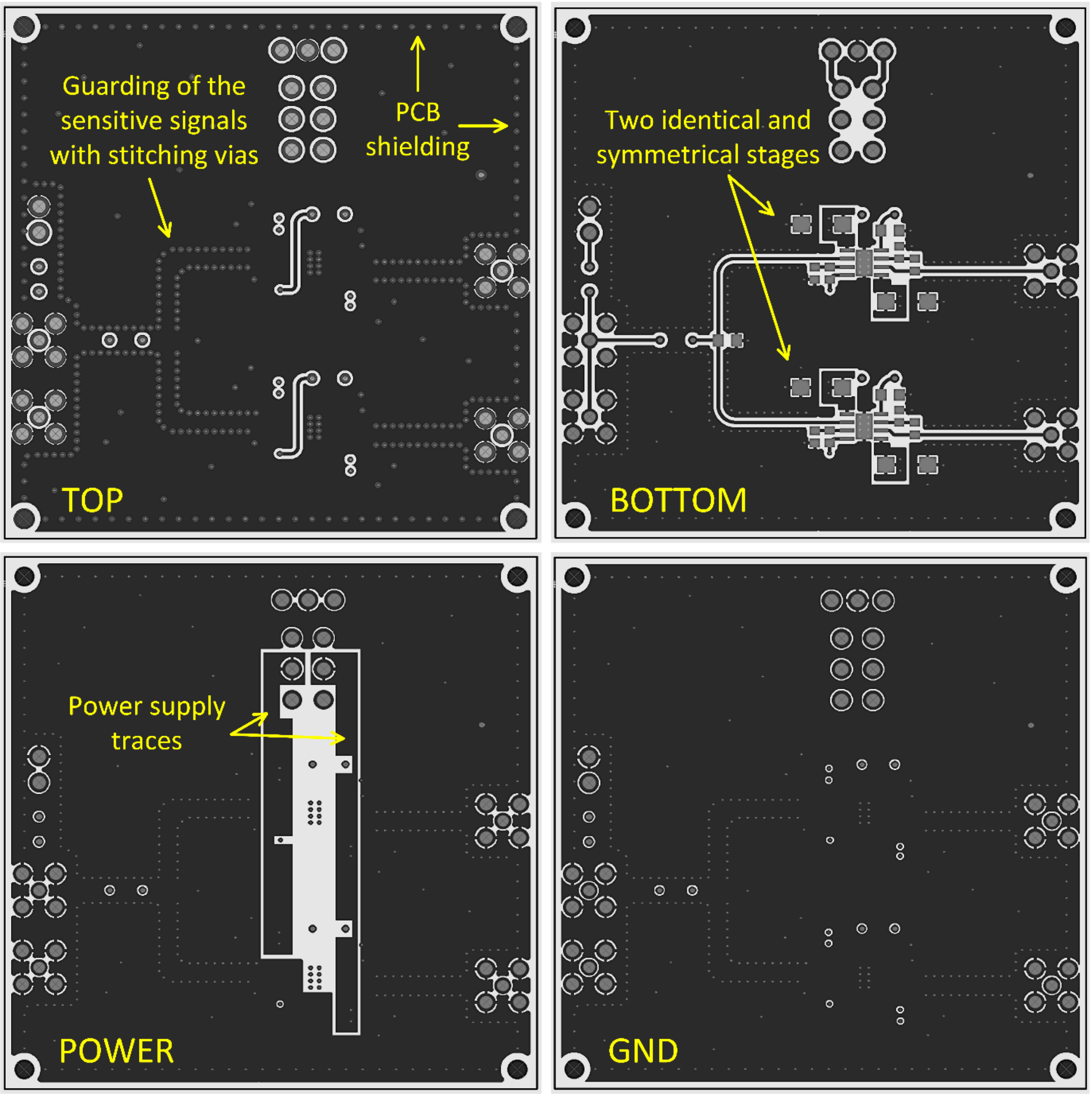

Figure S2. Ultra-low-noise amplifier

(**a**) Circuit diagram

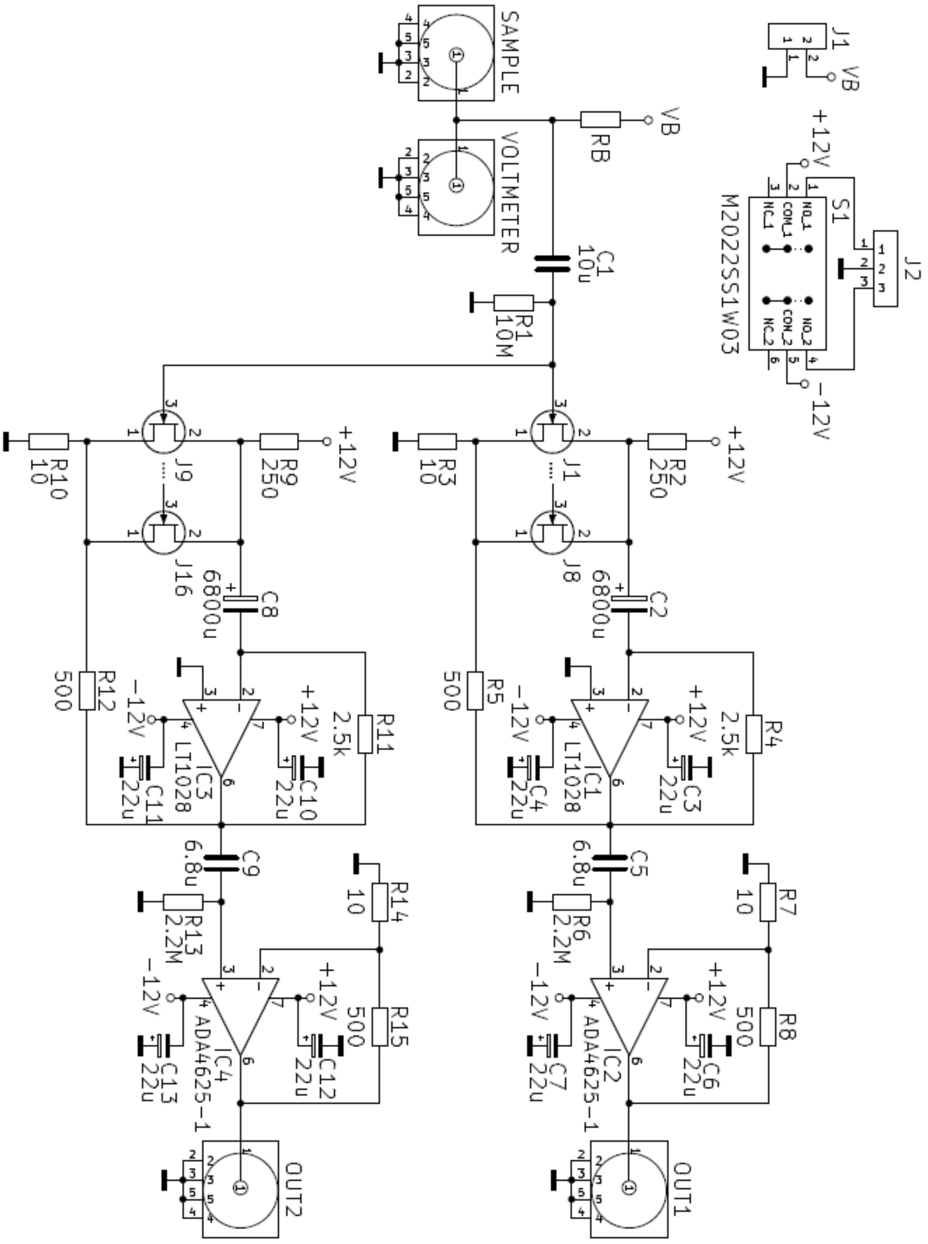

(**b**) PCB stack-up

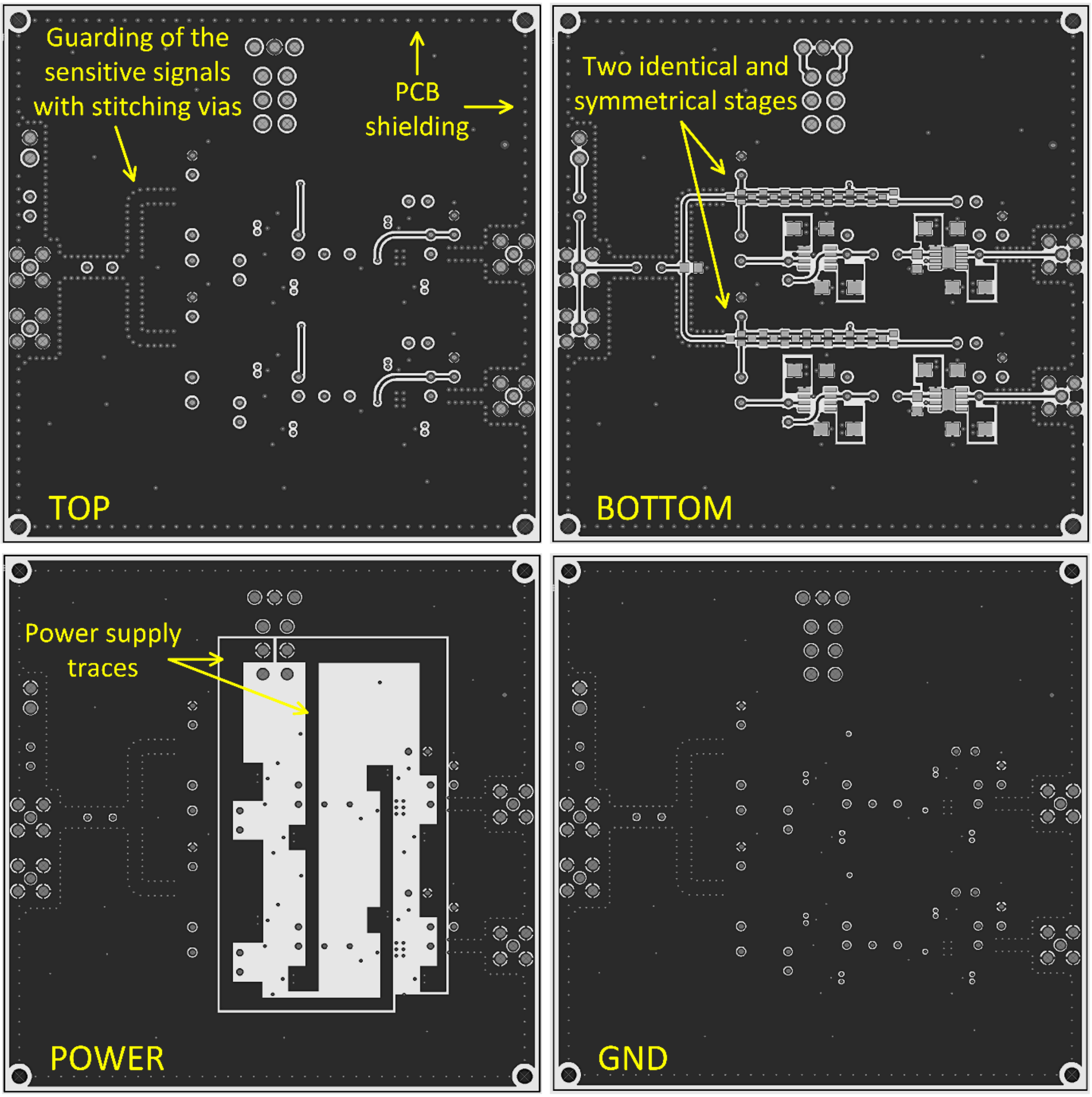

Figure S3. Wheatstone bridge and amplification circuit

(**a**) Circuit diagram

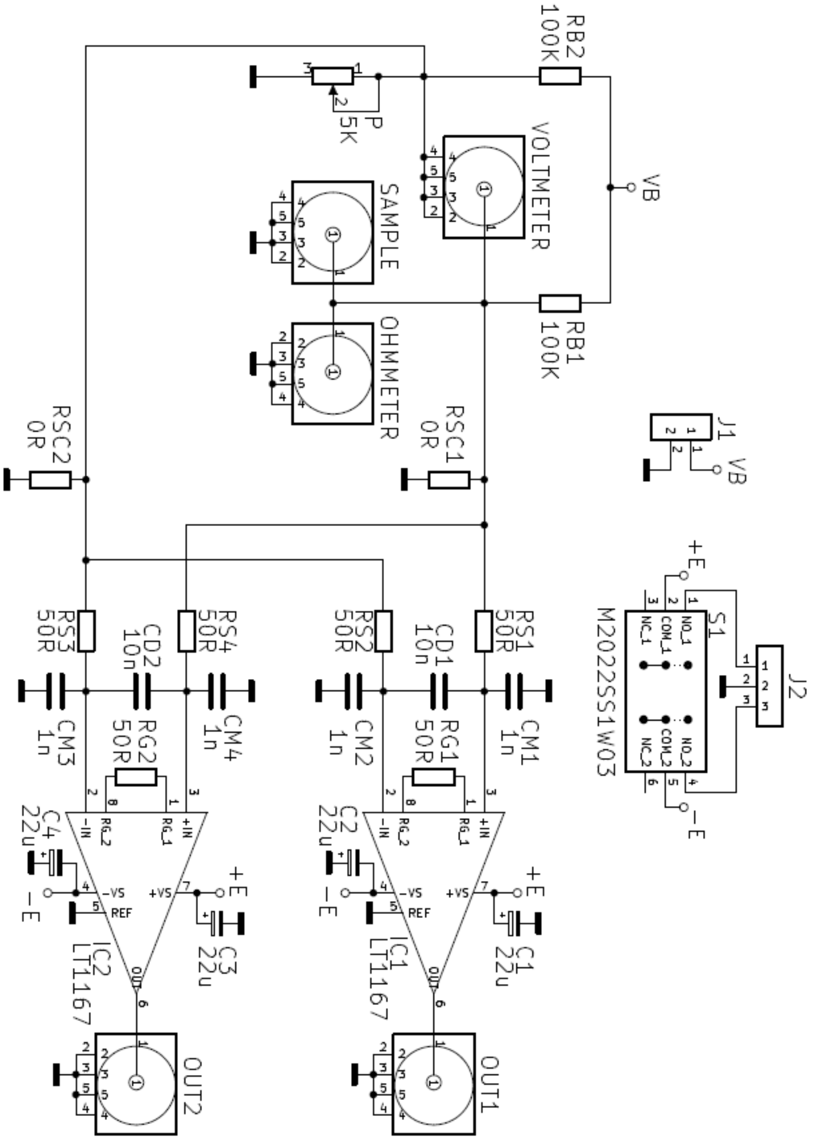

(**b**) PCB stack-up

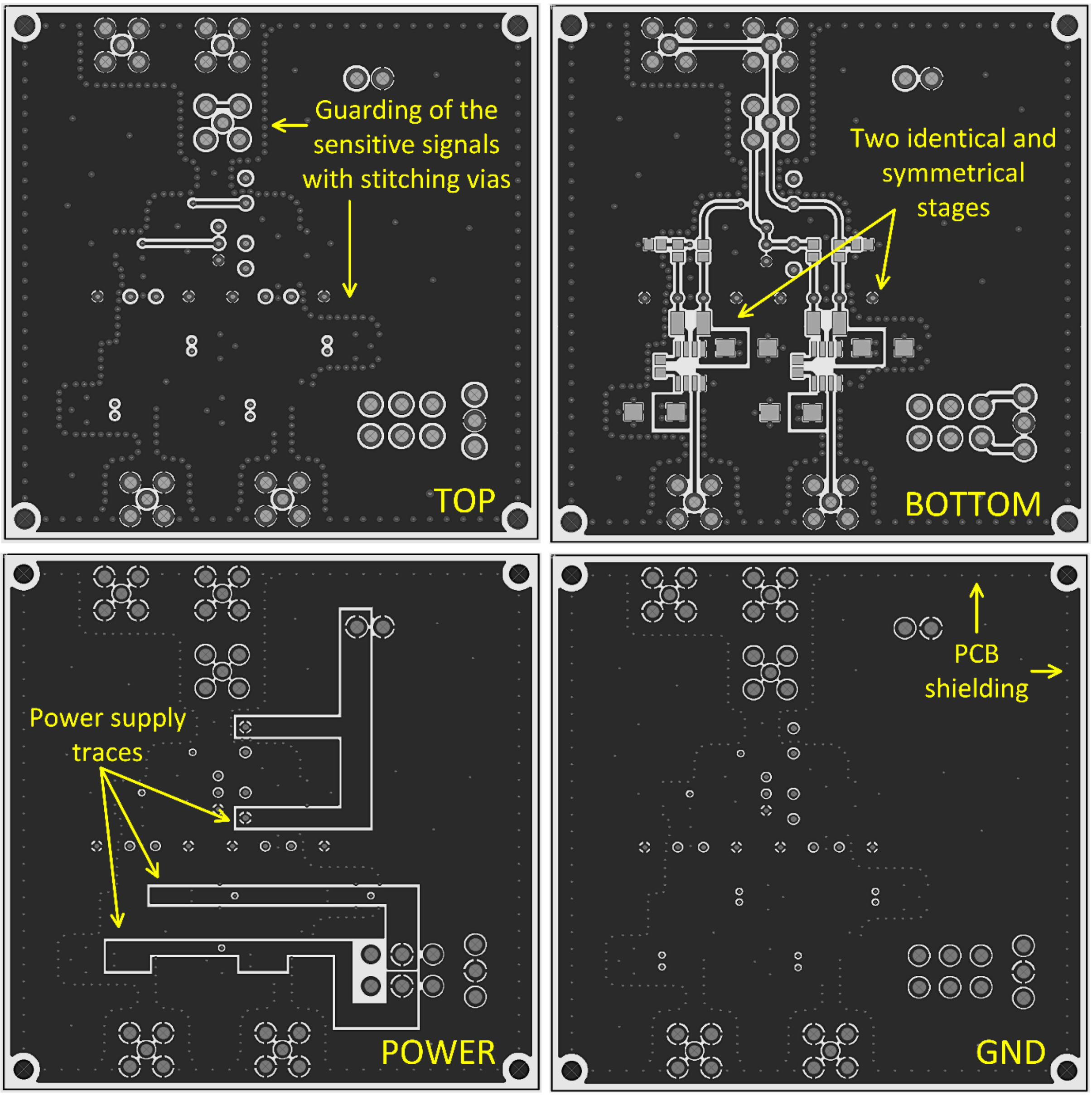

Supplement: Supplementary file 1 [file sensors-25-06182-s001.zip › sensors-3880687-supplementary.pdf]
